# Metal-Induced Stabilization and Activation of Plasmid Replication Initiator RepB

**DOI:** 10.3389/fmolb.2016.00056

**Published:** 2016-09-21

**Authors:** José A. Ruiz-Masó, Lorena Bordanaba-Ruiseco, Marta Sanz, Margarita Menéndez, Gloria del Solar

**Affiliations:** ^1^Molecular Biology of Gram-Positive Bacteria, Molecular Microbiology and Infection Biology, Centro de Investigaciones Biológicas (Consejo Superior de Investigaciones Científicas)Madrid, Spain; ^2^Biological Physical Chemistry, Protein Structure and Thermodynamics, Instituto de Química-Física Rocasolano (Consejo Superior de Investigaciones Científicas)Madrid, Spain; ^3^CIBER of Respiratory DiseasesMadrid, Spain

**Keywords:** HUH endonucleases, plasmid-encoded Rep proteins, metal-dependent catalytic activity, RepB thermostability, Mn^2+^ affinity

## Abstract

Initiation of plasmid rolling circle replication (RCR) is catalyzed by a plasmid-encoded Rep protein that performs a Tyr- and metal-dependent site-specific cleavage of one DNA strand within the double-strand origin (*dso*) of replication. The crystal structure of RepB, the initiator protein of the streptococcal plasmid pMV158, constitutes the first example of a Rep protein structure from RCR plasmids. It forms a toroidal homohexameric ring where each RepB protomer consists of two domains: the C-terminal domain involved in oligomerization and the N-terminal domain containing the DNA-binding and endonuclease activities. Binding of Mn^2+^ to the active site is essential for the catalytic activity of RepB. In this work, we have studied the effects of metal binding on the structure and thermostability of full-length hexameric RepB and each of its separate domains by using different biophysical approaches. The analysis of the temperature-induced changes in RepB shows that the first thermal transition, which occurs at a range of temperatures physiologically relevant for the pMV158 pneumococcal host, represents an irreversible conformational change that affects the secondary and tertiary structure of the protein, which becomes prone to self-associate. This transition, which is also shown to result in loss of DNA binding capacity and catalytic activity of RepB, is confined to its N-terminal domain. Mn^2+^ protects the protein from undergoing this detrimental conformational change and the observed protection correlates well with the high-affinity binding of the cation to the active site, as substituting one of the metal-ligands at this site impairs both the protein affinity for Mn^2+^and the Mn^2+^-driven thermostabilization effect. The level of catalytic activity of the protein, especially in the case of full-length RepB, cannot be explained based only on the high-affinity binding of Mn^2+^ at the active site and suggests the existence of additional, lower-affinity metal binding site(s), missing in the separate catalytic domain, that must also be saturated for maximal activity. The molecular bases of the thermostabilizing effect of Mn^2+^ on the N-terminal domain of the protein as well as the potential location of additional metal binding sites in the entire RepB are discussed.

## Introduction

The rolling circle replication (RCR) mechanism is used by transposons, small plasmids, phages, and viruses that replicate autonomously in a wide range of organisms, from prokaryotes to humans (Campos-Olivas et al., 2002). Plasmids that use this mechanism for their replication are termed RCR plasmids, and they are found in bacteria, archaea, and mitochondria (Novick, 1998; Khan, 2000; Ruiz-Masó et al., 2015). Initiation of plasmid RCR requires site-specific cleavage of one plasmid DNA strand within the double-strand origin (*dso*) of replication. This reaction is catalyzed by the metal-dependent endonucleolytic activity of the plasmid-encoded Rep protein, which yields a free 3′-OH end that serves as primer for initiation of the leading-strand synthesis by a host DNA polymerase. The initiator Rep also mediates the endonuclease and strand-transfer reactions that take place at the termination of the leading-strand replication process (Novick, 1998).

RCR plasmids have been classified into several replicon families based on sequence similarities at the Rep and *dso* level (del Solar et al., 1998; Khan, 2005; Ruiz-Masó et al., 2015). The replicon of pMV158, a small (5541 bp) multicopy promiscuous plasmid originally isolated from *Streptococcus agalactiae* and involved in antibiotic resistance spread, has been studied in depth and is considered as the prototype of a family of RCR plasmids isolated from several eubacteria (del Solar et al., 1993). RepB, the replication initiator protein of pMV158, carries out metal ion-dependent DNA cleavage and rejoining reactions as part of its replication function. Upon specific binding to the *dso*, RepB cleaves one strand of the DNA at a specific dinucleotide of the nick sequence (TACTACG/AC; / indicating the nick site) located on the apical loop of a hairpin formed by an inverted repeat (IR-I) (Moscoso et al., 1995; Ruiz-Masó et al., 2007). The nature of the cleavage reaction demands that the DNA substrate is in an unpaired configuration, which is achieved by IR-I hairpin extrusion on supercoiled DNA. *In vitro*, RepB contacts with its primary binding site (the *bind* locus) and with a region of the *nic* locus that includes the right arm of IR-I. Binding of RepB to the *bind* locus seems to facilitate binding of the protein to the *nic* locus, which promotes extrusion of the IR-I hairpin containing the substrate DNA to be cleaved (Ruiz-Masó et al., 2007). The nucleophilic attack on the scissile phosphodiester bond of the DNA is most likely exerted by the catalytic Tyr99 of RepB (Moscoso et al., 1997). Like other RCR Rep initiators from plasmids and bacteriophages, RepB lacks ATPase and helicase activities (de la Campa et al., 1990; Moscoso et al., 1995). Thus, apart from the DNA polymerase, other host proteins such as a superfamily 1 (SF1) DNA helicase and a single-stranded DNA (ssDNA)-binding protein are expected to be recruited to participate in the early stages of initiation and elongation.

RepB is a 210 amino acid polypeptide that is purified as a hexamer (RepB_6_, Ruiz-Masó et al., 2004). X-ray crystallography revealed the structure of full-length RepB_6_, which forms a toroidal homohexameric ring (Ruiz-Masó et al., 2004; Boer et al., 2009). Each RepB protomer comprises an N-terminal endonuclease domain, referred to as the origin binding domain (OBD), and a C-terminal oligomerization domain (OD) that forms a cylinder with a six-fold symmetry in the hexamer (Supplementary Figure 1). The conformational ensemble of RepB_6_ is characterized by a rigid cylindrical scaffold, formed by the ODs, to which the OBDs are attached as highly flexible appendages. The intrinsic flexibility allows RepB to adopt multiple conformational states and might be involved in the specific recognition of the *dso* (Boer et al., 2016). The N-terminal 131-residue OBD domain retains the DNA-binding and nuclease functions of the protein (Boer et al., 2009). This domain belongs to the superfamily of HUH endonucleases (in which U is a hydrophobic residue), which includes proteins of the Rep class, involved in replication of bacteriophages, plasmids, and plant and animal viruses, and of the Mob class, also known as relaxases, involved in the conjugal transfer of plasmid DNA (Ilyina and Koonin, 1992). The overall structure of the endonuclease domain of the HUH endonuclease superfamily is very similar despite the low level of sequence identity, and is characterized by a five-stranded antiparallel β-sheet flanked by a variable number of α-helices (Dyda and Hickman, 2003; Chandler et al., 2013). Moreover, the entire superfamily appears to follow a common endonucleolytic mechanism based on a catalytic Tyr and a divalent metal coordinated by a His cluster (Dyda and Hickman, 2003). The conserved HUH sequence motif, present in Rep and Mob proteins (Ilyina and Koonin, 1992), was confirmed as part of the metal binding site from structural data (Campos-Olivas et al., 2002; Hickman et al., 2002; Boer et al., 2009). Another conserved motif, designated UXXYUXK in Rep proteins, includes the catalytic Tyr (Ilyina and Koonin, 1992).

RepB OBD central β-sheet is flanked by helices α1 and α2 at one face, and by helix α3, which provides the catalytic residue Tyr99, and the short helix α4 at the opposite side. In addition, a Mn^2+^ cation is found close to Tyr99 in the active site (Supplementary Figure 1B). This metal ion is coordinated by five ligands, namely the RepB residues His39, Asp42, His55, and His57 (the latter two residues forming the HUH motif) and a single solvent molecule, in an octahedral-minus-one or square-based pyramidal geometry (Boer et al., 2009). All four RepB residues ligating the Mn^2+^ cation are placed in sequence motifs that are conserved in the Rep proteins of the pMV158 RCR plasmid family (del Solar et al., 1993), as is also the case with catalytic Tyr99 and with Tyr115, which hydrogen bonds to the Asp42 carboxyl group (Supplementary Figure 1). *In vitro*, only Mn^2+^ and Co^2+^, among various divalent cations tested, are able to promote RepB-mediated nicking-closing of supercoiled plasmid DNA (Boer et al., 2009). Thus, the presence of Mn^2+^ in the active site is consistent with these requirements. Although in DNA cleavage reactions where the hydroxyl group of a tyrosine or a serine acts as a nucleophile there is no apparent need of a metal cation for activation, the simultaneous presence of a tyrosine and a metal cation in the active site seems to be a common feature in the HUH endonucleases studied so far. In fact, it is generally accepted to attribute a structural role to the cation bound in the active site (Hickman et al., 2002; Larkin et al., 2005; Boer et al., 2006). In RepB, the Mn^2+^ ion probably interacts with the oxygen atoms of the scissile DNA phosphate, polarizing the bond and favoring the nucleophilic attack by the catalytic Tyr99 (Boer et al., 2009). The presence of additional divalent cation binding sites at the interface of OBD and OD domains has been reported for the C2 crystal structure of RepB_6_ (Boer et al., 2016).

Current structural information about full length Rep proteins from RCR plasmids is restricted to RepB, although the structure of a chimeric initiator Rep protein of staphylococcal plasmids belonging to the pT181 family has been recently solved (Carr et al., 2016). In addition, little information on biochemical and biophysical parameters has been reported for these proteins. In this work we have analyzed the effect of Mn^2+^ on both the thermostability and the catalytic activity of RepB. We demonstrate that the manganese cation strongly protects the protein from undergoing a thermal transition that otherwise takes place between 32 and 45°C. We also show that the conformational change associated with this transition is confined to the OBD and renders the protein catalytically inactive and unable to recognize the plasmid replication origin. Mn^2+^-driven thermostabilization of RepB most likely results from binding of the divalent cation to the active site of the protein, and is compatible with the metal affinity values obtained by isothermal titration calorimetry (ITC) for different protein variants. On the other hand, the analysis of the Mn^2+^ concentration dependence of the catalytic activity of the protein indicates that maximal activity of full-length RepB_6_ would require saturation of both the high affinity site in the active center and additional lower affinity site(s).

## Results

### Characterization of the RepB thermal transitions and their effects on the protein activity

**Figure 1:**
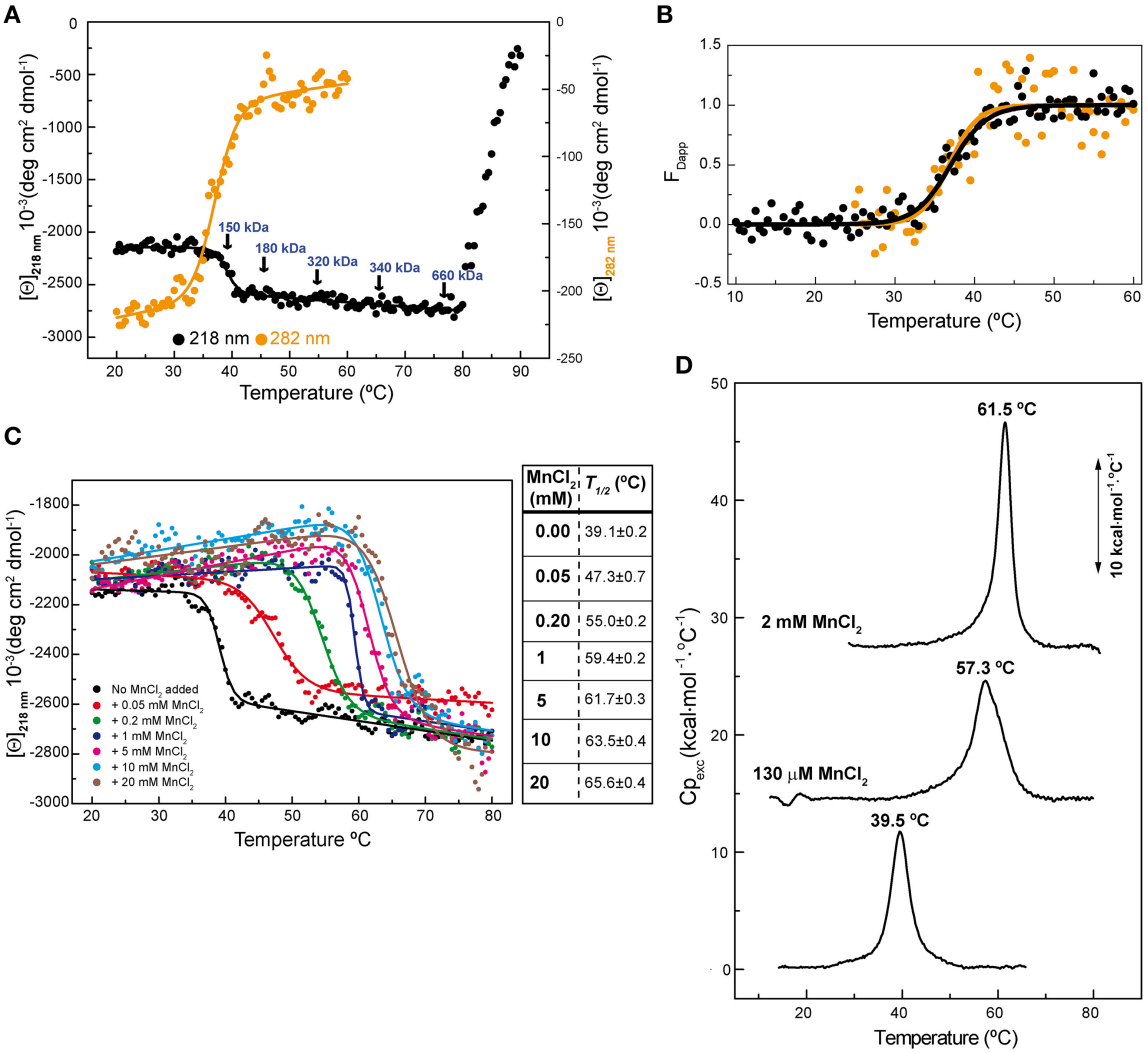
**RepB_6_ low-temperature transition involves global changes in the protein structure**. **(A)** Temperature-induced conformational changes of RepB_6_ as monitored by the variation of the ellipticity at 218 and 282 nm ([Θ] represents protein molar ellipticity). Samples heated at the indicated temperatures (arrows) were analyzed by analytical ultracentrifugation (sedimentation equilibrium), and the estimated average molecular masses are displayed on the protein CD thermal profile. **(B)** Apparent fraction of modified protein (F_Dapp_) calculated from the transition curves registered at 218 nm (•) and 282 nm (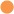) between 10 and 60°C. The solid line shows the fit of Equation (1) to F_Dapp_ values. **(C)** Temperature transition curves of RepB_6_ (12 μM) in the presence of increasing concentrations of MnCl_2_ (indicated inside the graph) measured by CD at 218 nm. The table shows the apparent half-transition temperatures of RepB_6_ derived from fit of Equation (1) to the figure experimental curves (solid lines). **(D)** DSC profile of the first thermal transition of RepB_6_ (30 μM) monitored in the absence and in the presence of 130 μM or 2 mM Mn^2+^. The position of the maximum of the heat capacity function (*T*_*m*_) is indicated.

Previous circular dichroism (CD) studies on hexameric RepB_6_ revealed the presence of a temperature-induced irreversible transition between 32 and 45°C leading to a small, but significant, increase of the protein α-helical content, whereas a second transition occurring above 80°C resulted in RepB precipitation (Ruiz-Masó et al., 2004). We now show that the first transition also induced a decline in the ellipticity signal at 282 nm (Figure 1A), indicative that RepB_6_ tertiary/quaternary structure was also modified, and that the transition advance estimated from the CD thermal profiles at 282 and 218 nm fully overlapped (Figure 1B). The irreversibility of such conformational change allowed us to analyze, by analytical ultracentrifugation, the oligomerization state of RepB_6_ heated to different temperatures in the range from 25 to 75°C. As indicated in Figure 1A, the average molecular weight remained close to that of the hexamer (145.5 kDa) up to the end of the first transition (M_app_/M_0_ = 1.2 at 45°C; M_0_ being the hexamer molecular weight). However, a clear increase in the oligomerization state was observed as the temperature was further increased, followed by the protein precipitation above 80°C.

**Figure 2:**
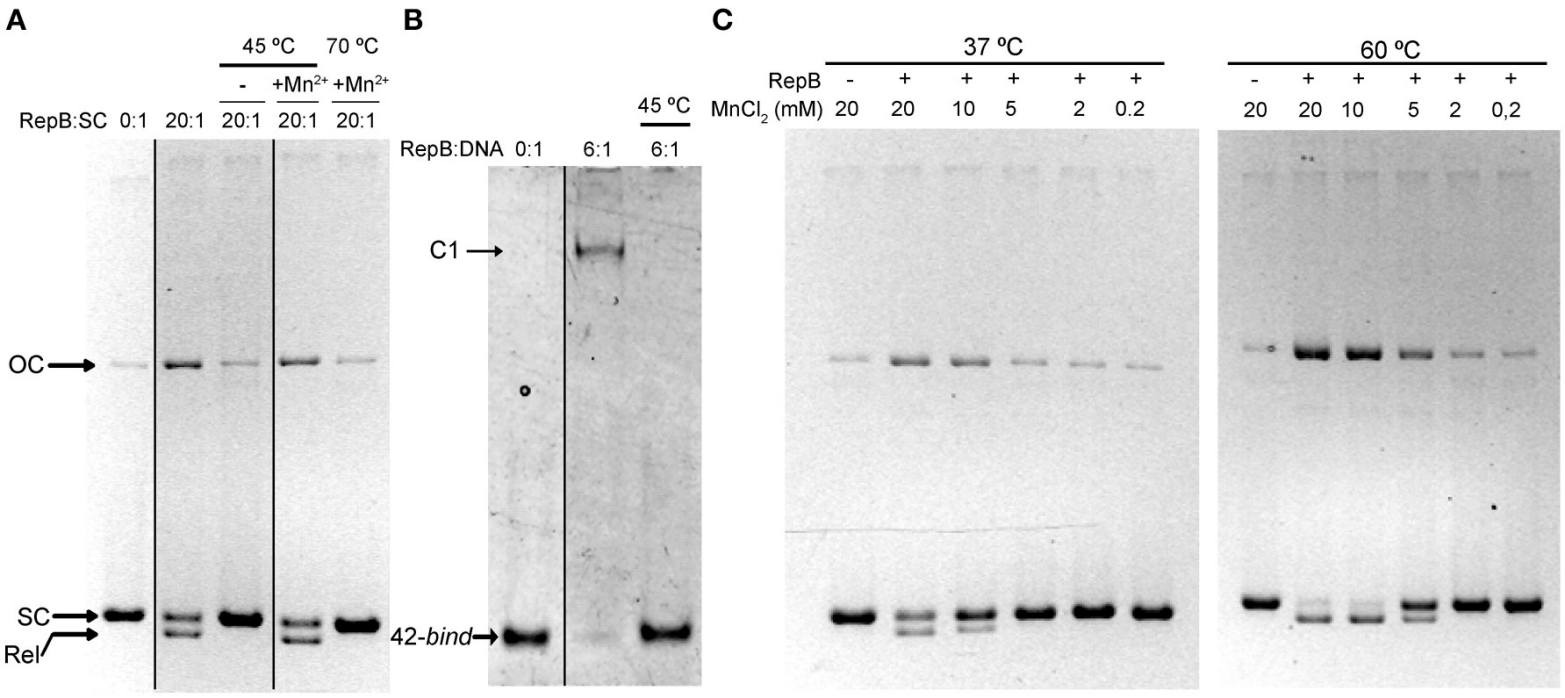
**The presence of Mn^2+^ activates RepB and protects it against thermal inactivation. (A)** Activity assays on supercoiled DNA. Samples of RepB unheated, heated to 45°C in the absence of Mn^2+^, or heated to 45 or 70°C in the presence of 20 mM Mn^2+^ were mixed, at the indicated molecular ratios, with 6.7 nM of pMV158 supercoiled DNA and then incubated for 30 min at 37°C in the presence of 20 mM MnCl_2_. The supercoiled (SC), closed relaxed circles (Rel), and open circular (OC) plasmid forms were separated by electrophoresis on agarose gels containing ethidium bromide. Images from different parts of the same gel have been grouped and indicated by dividing lines. **(B)** RepB_6_ recognition of the *bind* DNA measured by EMSA. Unheated or 45°C-heated samples of RepB_6_ were mixed, at the indicated molecular ratios, with 0.4 μM of a 42-bp DNA fragment containing the three 11 bp-direct repeats that constitute the *bind* region of the pMV158 *dso*. Positions of the free (42-*bind*) and complexed (C1) DNA are indicated. Images from different parts of the same gel have been grouped and indicated by dividing lines. **(C)** Supercoiled pMV158 DNA (6.7 nM) was incubated, either at 37°C or at 60°C, with the indicated concentrations of Mn^2+^, in the absence (−) or in the presence (+) of purified RepB_6_ (RepB:pMV158 DNA molecular ratio of 20:1).

**Figure 3:**
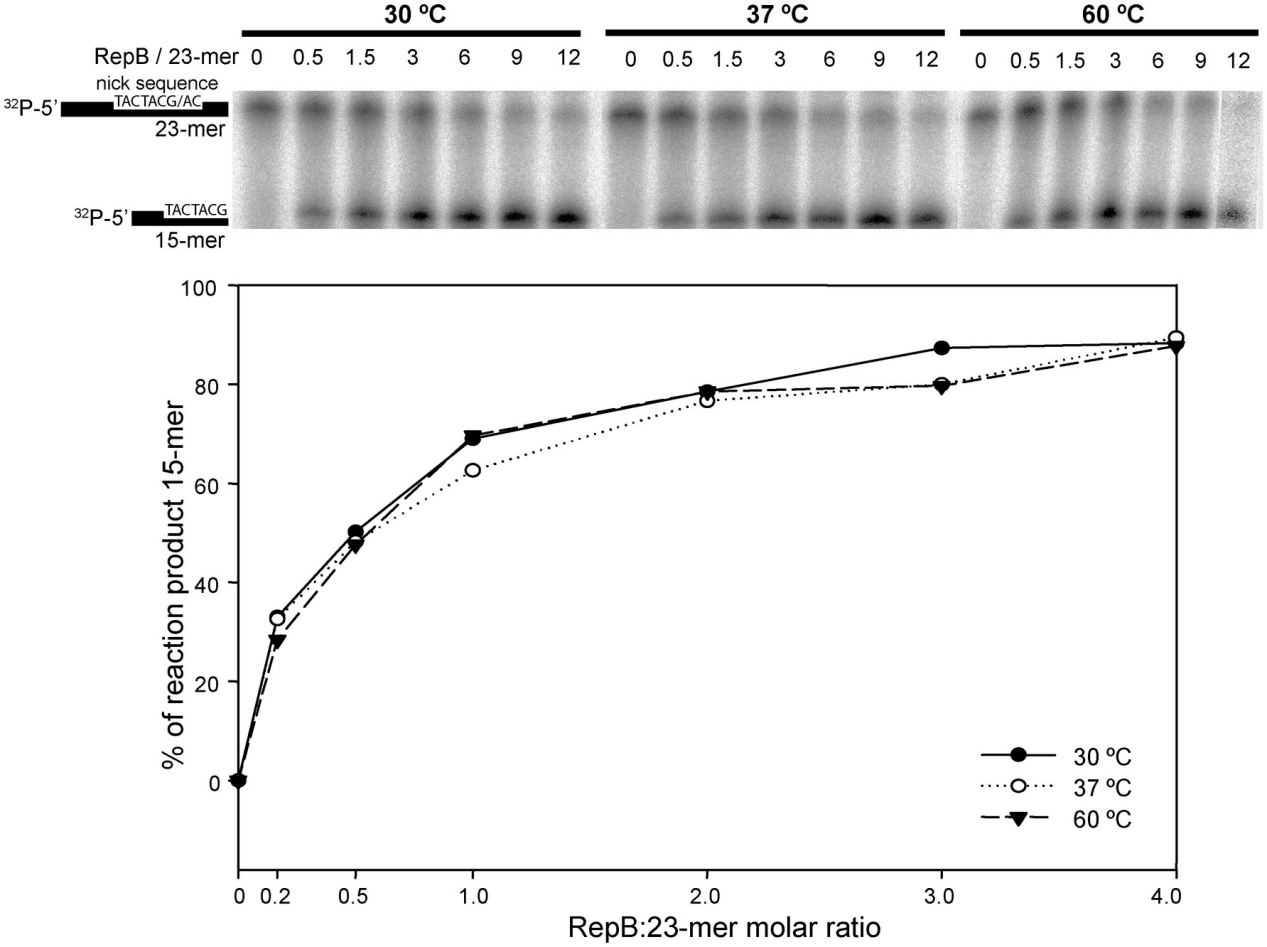
***In vitro* cleavage activity of RepB_6_ on ssDNA oligos at different temperatures**. A 23-mer oligo substrate (100 nM), radioactively labeled in 5′, was incubated with RepB_6_ at three different temperatures (30, 37, and 60°C) in the presence of 20 mM of MnCl_2_ and at the indicated protein:oligo substrate molar ratios. The products were separated on 20% PAA, 8M urea denaturing gels (upper part). Nicking activity of RepB_6_ was quantified as the percentage of 15-mer product formed (lower part).

To investigate the effect of the first thermal transition on RepB_6_ activity as RCR initiator, we tested the nicking/closing and DNA binding capacities of RepB_6_ after being heated or not to 45°C. The results showed that the protein heated to 45°C was unable to relax the supercoiled (sc) cognate plasmid DNA (Figure 2A) and had also lost its ability to bind to the target dsDNA (Figure 2B). In contrast with this, the RepB_6_ nicking/closing activity on scDNA was maximal at 60°C in the presence of 10–20 mM MnCl_2_, whereas it decreased to about 50% when the reaction was carried out at the same Mn^2+^ concentrations but at 37°C, the optimal growth temperature of the pMV158 pneumococcal host (Moscoso et al., 1995; Figure 2C). It is noteworthy that the enhancement of RepB_6_ activity at 60°C is restricted to sc plasmid DNA and was not observed on ssDNA substrates unable to form the IR-I cruciform (Figure 3). Therefore, the higher activity at 60°C is most likely due to the high temperature facilitating the extrusion of the cruciform that renders the nick sequence a single-stranded substrate. Be that as it may, preservation of the activity at 60°C required a factor specifically present in the reaction mixture that protected the protein from the thermal inactivation. Mn^2+^ cations were next shown to account for this role, as the presence of 20 mM MnCl_2_ during RepB_6_ heating to 45°C prevented its inactivation and kept intact its endonuclease (Figure 2A) and DNA binding activities (not shown). Regardless of the presence of MnCl_2_, the sample heated up to 70°C was completely inactive (Figure 2A). To explore the influence of Mn^2+^ on the structure and thermal stability of RepB_6_, we first compared the CD spectra (far- and near-UV regions) of the protein in the presence and in the absence of MnCl_2_. Their coincidence indicated that no significant changes occurred in either the secondary structure or the tertiary/quaternary structure upon binding of Mn^2+^ (not shown). Next we carried out thermal denaturation experiments in the presence of Mn^2+^ at concentrations ranging from 0.05 to 20 mM and the first thermal transition was assessed by monitoring RepB_6_ ellipticity at 218 nm. The results showed a strong stabilization of RepB_6_ by Mn^2+^, shifting the apparent half-transition temperature (*T*_1/2_) by around 25°C (from ~39 to ~66°C) at the maximum concentration of MnCl_2_ tested (Figure 1C). In contrast, MgCl_2_ or CaCl_2_ addition had no effect on RepB_6_ stability (not shown). The steepest variation of *T*_1/2_ occurred below 1 mM and the progression of *T*_1/2_ at higher ligand concentrations followed the trend expected for ligand binding domains (Brandts et al., 1989).

The influence of Mn^2+^ in RepB_6_ structural stability was also examined by differential scanning calorimetry (DSC; Figure 1D). In the absence of cation, the thermogram shows a peak with a transition temperature (*T*_*m*_) of 39.5°C, very close to the *T*_1/2_ obtained for the first CD transition, and a transition enthalpy change of 71 kcal/mol of protomer, which supported a protein denaturation event. Above 80°C the baseline dropped drastically due to RepB_6_ precipitation, in agreement with CD results. The visible peak was drastically shifted to higher temperatures upon Mn^2+^ addition (57.3 and 61.5°C for 130 μM and 2 mM of Mn^2+^, respectively; Figure 1D). The cation addition also increased the transition enthalpies to 89 kcal/mol (130 μM Mn^2+^) and 129 kcal/mol (2 mM Mn^2+^).

### The first thermal transition is confined to the catalytic domain

**Figure 4:**
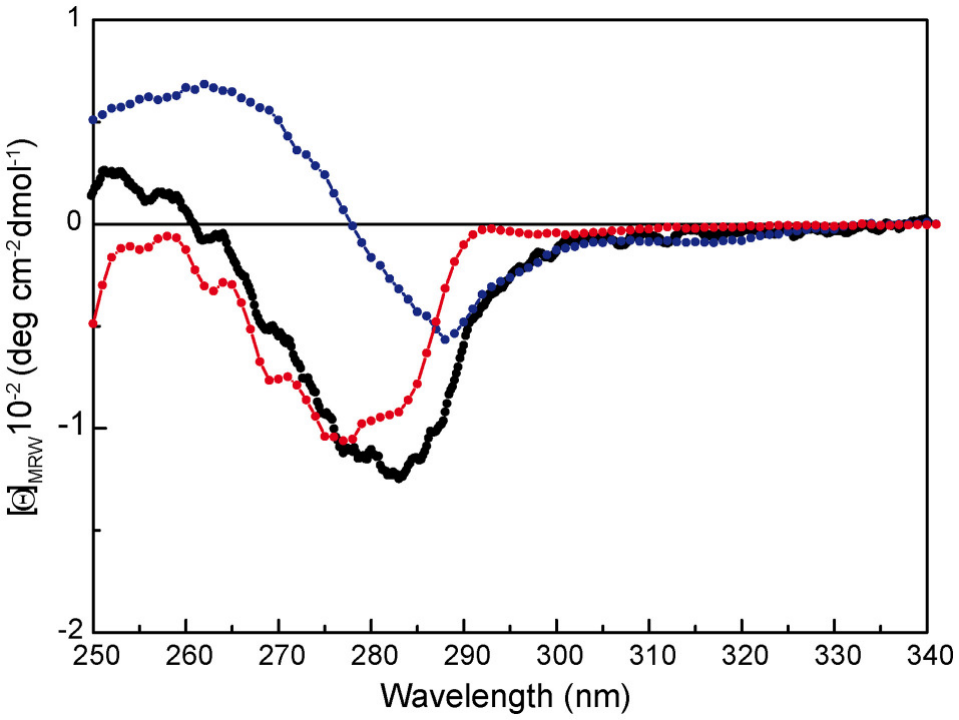
**Near-UV CD spectrum of RepB (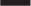), OBD (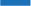), and OD (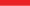) separate domains**. [Θ] represents the mean residue ellipticity. For comparison, the OBD and OD spectra have been weighted by the fractional contribution of their amino acids to the complete protein sequence.

To investigate whether the first thermal transition affects a particular protein domain, we analyzed the thermal stability of the separate RepB domains, purified from *Escherichia coli* as described (Boer et al., 2009). The N-terminal OBD, which has been shown by analytical ultracentrifugation to be in a monomeric state, contains the endonuclease and DNA binding activities, and retains these abilities when separated from the C-terminal OD, which maintains its hexameric structure (Boer et al., 2009, 2016). Of note, the near-UV CD spectra of the separate domains correlate fairly well with that of RepB_6_ (i.e., the RepB_6_ spectrum approximately matches the curve obtained by addition of the spectra of the separate domains weighted by the fractional contribution of their amino acid number to the complete protein), evidencing the conservation of tertiary/quaternary structure in both domains (Figure 4).

Thermal stability of OBD and OD was studied by CD spectroscopy following the procedure used for RepB_6_. The CD thermal profile of OD at 218 nm showed that the oligomerization domain suffered a single thermal transition when the temperature was raised above 78°C, which correlated with the observable precipitation of the sample and the reduction of the spectrum intensity (Supplementary Figure 2). Although the three-dimensional structure of RepB_6_ did not reveal a metal binding site specific of the OD (Boer et al., 2009), we decided to assess the effect of Mn^2+^ in the stability of the domain. The presence of 1 mM MnCl_2_ during the heating of the sample delayed the thermal transition about 5°C. This effect was not specific of Mn^2+^ and MgCl_2_ produced the same stabilization (not shown).

**Figure 5:**
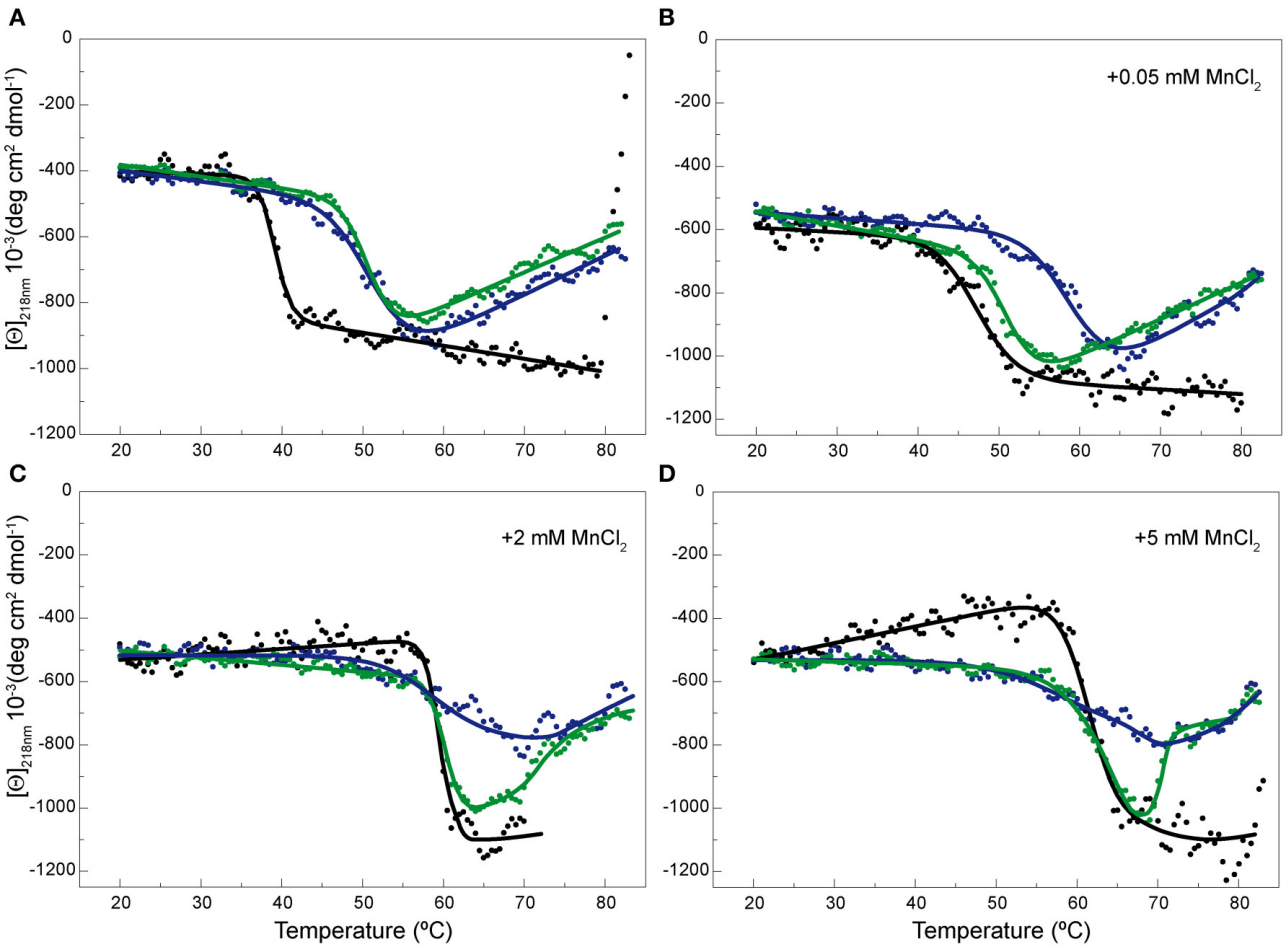
**Temperature-induced changes in the secondary structure of OBD and OBD^D42A^**. Temperature transition curves of RepB_6_ (•), OBD (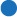), and OBD^D42A^ (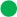) measured by CD at 218 nm in the absence of MnCl_2_ **(A)** or in the presence of 0.05 **(B)**, 2 **(C)**, and 5 **(D)** mM of MnCl_2_ ([Θ] represents the protein molar ellipticity). The CD thermal profile of RepB_6_ has been shifted along the ordinate axis to facilitate the comparison with those OBD and OBD^D42A^. In the case of OBD and OBD^D42A^, the continuous lines represent an average of the experimental data. Measurements were carried out at 12 μM RepB_6_ and 19 μM OBD or OBD^D42A^.

Prior to its thermal characterization, purified OBD, which carried a His-tag, was subjected to an extra-chelating treatment aimed to eliminate trace amounts of divalent cations from the purification steps. The OBD thermal profile at 218 nm shows a single irreversible structural change that takes place with a *T*_1/2_ of ~51.5°C. The structural change increased by 66% the ellipticity value at 218 nm, though the intensity of the whole far-UV spectrum decreased when the temperature was raised above 60°C due to OBD precipitation (Figure 5A and Supplementary Figure 3A). Of note, the first structural change of RepB_6_ has the same magnitude in protomer molar ellipticity units than the transition of OBD, whereas the cooperativity of the process appears to be somewhat different (Figure 5A). The presence of MnCl_2_ during the heating of the OBD sample stabilized the domain structure, increasing by around 13°C the on-set of the thermal transition at 5 mM MnCl_2_ (OBD precipitation after denaturation hampered the estimation of *T*_1/2_ values above 2 mM Mn^2+^; Figure 5).

Contribution of the active site cation to the stability and the catalytic activity of OBD was evaluated by replacing the acidic residue Asp42, involved in Mn^2+^ binding to the active center, by alanine. As for OBD, the protein mutant was treated with EDTA, prior to its equilibration in CD buffer, to eliminate any trace of divalent cations from the purification steps. The far-UV CD spectra of OBD and OBD^D42A^ acquired at 20°C were very similar, if not identical (Supplementary Figure 3), and the presence of MnCl_2_ did not modify the spectra (not shown). Generation of the mutant OBD^D42A^ resulted in a protein variant whose thermal stability was comparable to that of the wild type domain in the absence of Mn^2+^. In fact, both the magnitude of the ellipticity change at 218 nm and the *T*_1/2_ of OBD^D42A^ and OBD (50.5 and 51.5°C, respectively) were similar (Figure 5A). Despite removal of a Mn^2+^ ligand in the active site, OBD^D42A^ still has the capacity to bind Mn^2+^, as shown by the ability of Mn^2+^ to up shift the thermal denaturation of the mutant domain (Figures 5B–D). However, at low MnCl_2_ concentrations the transition shift was lower in the mutant, probably due to the loss of one of the metal ligand and the consequent Mn^2+^ affinity decrease (Figure 5B). Together, these results evidenced that first thermal transition displayed by RepB_6_ corresponds to the OBD catalytic domain, and that OBD is the receptor of Mn^2+^ cations accounting for RepB_6_ stabilization. Besides, the magnitude of the enthalpy change associated to this transition strongly indicates that it implies OBD denaturation.

### Determination of RepB_6_-Mn^2+^ binding affinity by ITC

**Figure 6:**
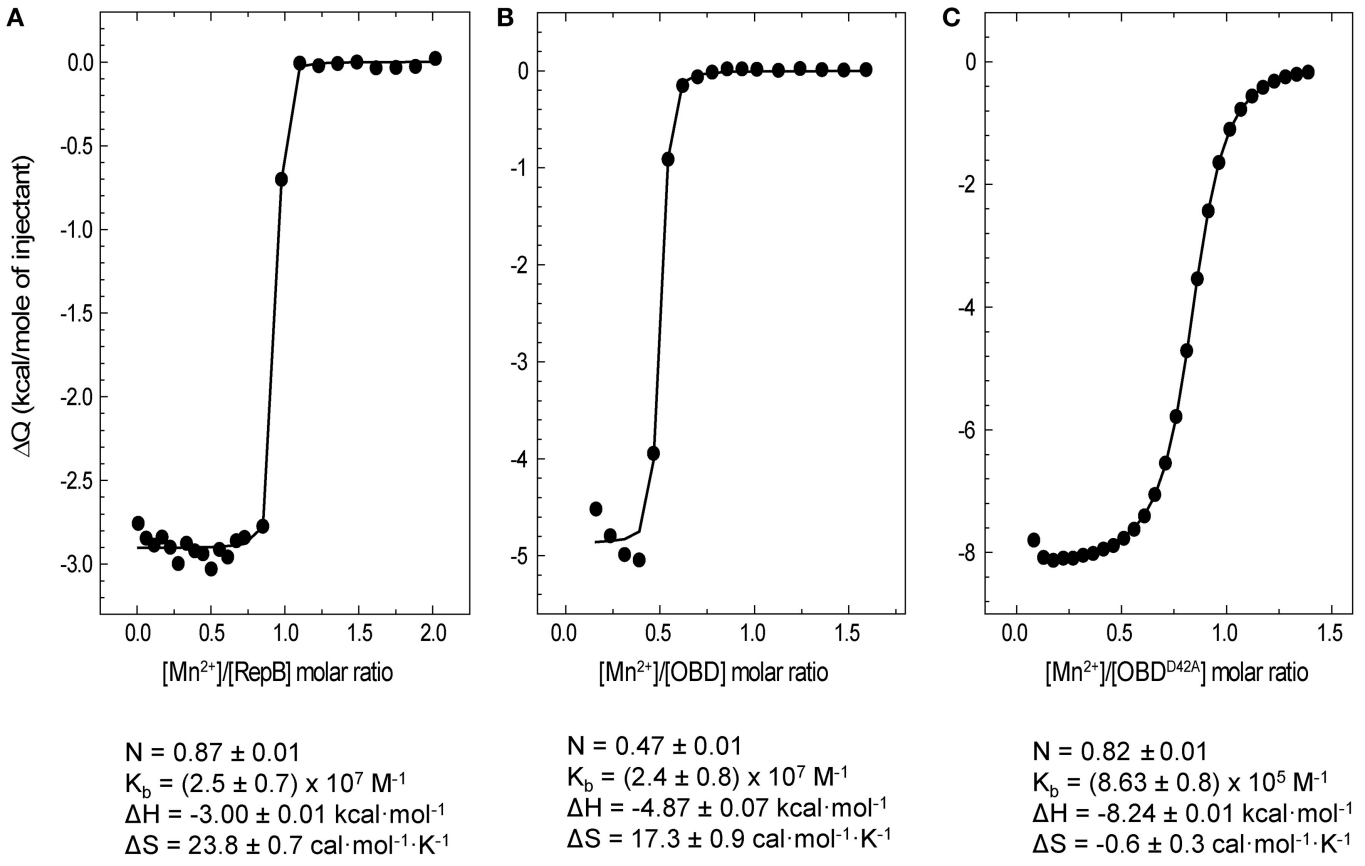
**ITC analysis of Mn^2+^ binding to RepB_6_, OBD, and OBD^D42A^**. Symbols represent the heat released by mole of Mn^2+^ injected as a function the Mn^2+^/protein molar ratio measured at 25°C in ITC buffer. Titrations were performed by adding 1 mM MnCl_2_ to RepB_6_**(A)**, OBD **(B)**, or OBD^D42A^ **(C)** proteins at concentrations ranging from 95 to 119 μM. The binding parameters derived from the fit of the single site binding model to the experimental curve are shown at the bottom and the corresponding theoretical curves are depicted as solid lines.

The affinity of RepB_6_, OBD and OBD^D42A^ for Mn^2+^ was examined by ITC. Titrations were performed at 25°C. The analysis of the binding isotherms (Figure 6) showed that each protomer of RepB_6_ binds one Mn^2+^ cation with high affinity (*K*_b_ = (2.5 ± 0.7) × 10^7^ M^−1^; N = 0.87 ± 0.01 sites/protomer) and a binding enthalpy of −3.00 ± 0.01 kcal mol^−1^. The affinity of Mn^2+^ for the isolated OBD domain was rather similar (*K*_b_ = (2.4 ± 0.8) × 10^7^ M^−1^), but the number of titrable sites was drastically reduced (N = 0.47 ± 0.01 sites/monomer). In contrast, the *N*-value of 0.82 obtained for the OBD^D42A^-Mn^2+^ complex compared well with that of the complete protein, and the binding affinity was reduced to about one-thirtieth (*K*_b_ = (8.63 ± 0.08) × 10^5^ M^−1^). As shown by the thermodynamic parameters displayed in Figure 6, Mn^2+^ binding to RepB active site is entropically driven, which suggests that primarily occurs through electrostatic interactions and possible removal of bound solvent molecules from the binding interface. However, substitution of Asp42 by alanine made the enthalpy of binding −3.37 kcal mol^−1^ more favorable but almost canceled the entropic contribution, evidencing that Mn^2+^ binding to OBD^D42A^ implies hydrogen bond formation and/or an entropically unfavorable reorganization of the domain structure.

The high affinity of OBD and OBD^D42A^ for Mn^2+^ is consistent with the strong metal-dependent stabilization observed in the CD thermal profiles and the DSC thermograms (Figures 1, 5). On the other hand, the reduced binding capacity of OBD could denote a high proportion of non-functional domain or, alternatively, previous occupance of Mn^2+^ binding sites. This later possibility could also explain the higher stability of OBD in CD buffer without Mn^2+^ compared to RepB_6_.

Titration of RepB_6_ with 1 mM MgCl_2_ produced neither heat uptake nor relase, and supplementation of ITC buffer with 2 mM MgCl_2_ did not changed Mn^2+^ affinity for RepB_6_ (not shown). These results, together with the failure of MgCl_2_ to stabilize the OBD domain, strongly indicates that Mg^2+^ cannot substitute Mn^2+^ at the active site.

### The effect of metal binding on OBD and RepB_6_ catalytic activity

**Figure 7:**
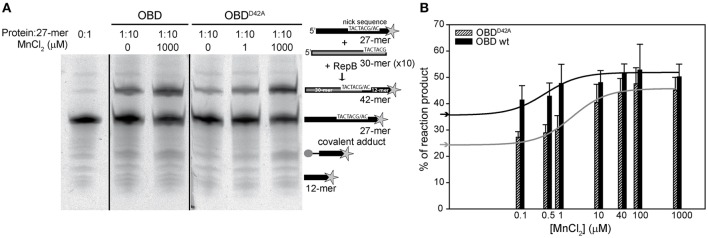
**Nicking and strand-transfer activity of OBD and OBD^D42A^ on ssDNA oligos in the presence of different metal concentrations. (A)** Reaction product pattern generated by the nicking and strand-transfer activities of OBD and OBD^D42A^ on ssDNA oligos at the indicated protein:oligo substrate molar ratio and different concentrations of MnCl_2_. The 27-mer oligo substrate (500 nM), labeled in 3′ with the fluorescent dye Cy5 (indicated by a star), and a 10-fold molar excess of the unlabeled 30-mer oligo were incubated with the protein at 37°C for 1 min. The resultant fluorescent oligos were analyzed by electrophoresis in 20% PAA, 8 M urea denaturing gels and visualized with the aid of FLA-3000 (FUJIFILM) imaging system. A schematic description of the different reaction products is depicted on the right side of the gel image. To compare the reactions products generated by the activity of OBD and OBD^D42A^ the images from different gels acquired and processed under the same conditions have been grouped and indicated by dividing lines. **(B)** Vertical bar graph comparing the percentage of reaction products rendered by OBD and OBD^D42A^ due to the addition of the indicated concentrations of MnCl_2_. The assays were performed as depicted in panel A with a protein:oligo substrate molar ratio of 1:10. Vertical bars represent the average value of three different experiments. Errors bars represent standard deviations. The activity increases of OBD and OBD^D42A^ were fitted by nonlinear regression (solid line) to a ligand binding model assuming a single class of binding site for Mn^2+^. The best fitting values for the apparent dissociation constant for OBD and OBD^D42A^ were 0.5 ± 0.3 and 2.6 ± 0.6 μM, respectively. The percentage of reaction products measured in the absence of MnCl_2_ added for OBD (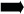) and OBD^D42A^ (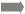) is indicated on the y-axis.

RepB_6_ and OBD are able to catalyze the joining of the 5′-phosphate end of the cleavage reaction product with a new 3′-OH end (Moscoso et al., 1995). The effect of different divalent metals on the activity of OBD and RepB_6_ on single-stranded oligonucleotides (oligos), as well as the influence of the D42A mutation, was assessed by performing cleavage and strand-transfer assays. For these experiments, OBD and OBD^D42A^ proteins were subjected to the extra chelating treatment indicated above after removal of their His-tags. In order to reveal the total fraction of reaction products, the reaction mixtures contained 10 pmol of a Cy5 3′-labeled 27-mer substrate carrying the specific nick sequence, and a 10-fold molar excess of an unlabeled 30-mer that provided the 3′-OH substrate for strand transfer, thus avoiding re-joining of the 27-mer oligo. The mixture of oligos was treated with OBD or OBD^D42A^ as indicated in the Experimental Procedures and, subsequently, the reaction products were analyzed by electrophoresis in PAA-urea sequencing gels. Cleavage and strand-transfer activities resulted in the generation of two new fluorescent bands corresponding to 12- and 42-mer products, respectively. In addition, incubation of the samples with SDS and proteinase K, used to stop the reaction, allowed the detection of a covalent complex between OBD and the 12-mer oligo, which appeared as a third fluorescent band corresponding to a small peptide linked to the 5′ end of the 12-mer oligo (Figure 7A). The fraction of labeled DNA in each of the three reaction products was calculated and used to determine the protein total activity. Under these conditions of substrate excess, the strand-transfer activity of OBD and OBD^D42A^ was prevalent regardless of the Mn^2+^ concentration and of the protein variant used, and the main reaction product was the 42-mer (Figure 7A). The effect of adding increasing concentrations of MnCl_2_ on the level of substrate conversion by OBD or OBD^D42A^ is displayed in Figure 7B. It should be noted that the catalytic activity of OBD was fully dependent on the metal ion, as deduced from the absence of reaction products in the presence of 10 mM EDTA (not shown). However, in the absence of EDTA and at 0.1 μM of MnCl_2_, the lowest metal cation concentration added, the reaction products amounted to ~36 and 41% of the 27-mer total added, respectively; that is, ~66–76% of the maximal activity, which was reached at around 40 μM MnCl_2_. These values reflect the high binding affinity of OBD for Mn^2+^, for which an apparent dissociation constant of 0.5 ± 0.3 μM was estimated assuming that the activity increase above the background reflected the saturation of the cation available sites. The catalytic activity of OBD^D42A^ also augmented upon increasing MnCl_2_ concentrations. At 0.1 μM Mn^2+^ the percentage of reaction products (~25% of the initial substrate) was, again, very close to the value with no MnCl_2_ added, and represented a 51% of substrate conversion under conditions of maximal activity (Figure 7B). The apparent dissociation constant for the Mn^2+^ cations accounting for this activity increase (2.6 ± 0.6 μM) was around five-folds higher than for wild-type OBD. The Mn^2+^ apparent dissociation constant inferred for OBD^D42A^ from the activity assays (~2.6 μM) matched quite well the value of *K*_d_ (~2 μM) obtained by extrapolation of the ITC constant to 37°C, whereas that of OBD (~0.5 μM) was around nine-fold higher than the ITC-derived value (*K*_d,37°C_ ≅57 nM). This apparent discrepancy was probably due to the errors of the activity measurements and to the small net increment of OBD activity at saturation by Mn^2+^ with relation to the background without Mn^2+^, whose high value likely reflects the capture of Mn^2+^ traces present in the reaction mixture by the active site.

**Figure 8:**
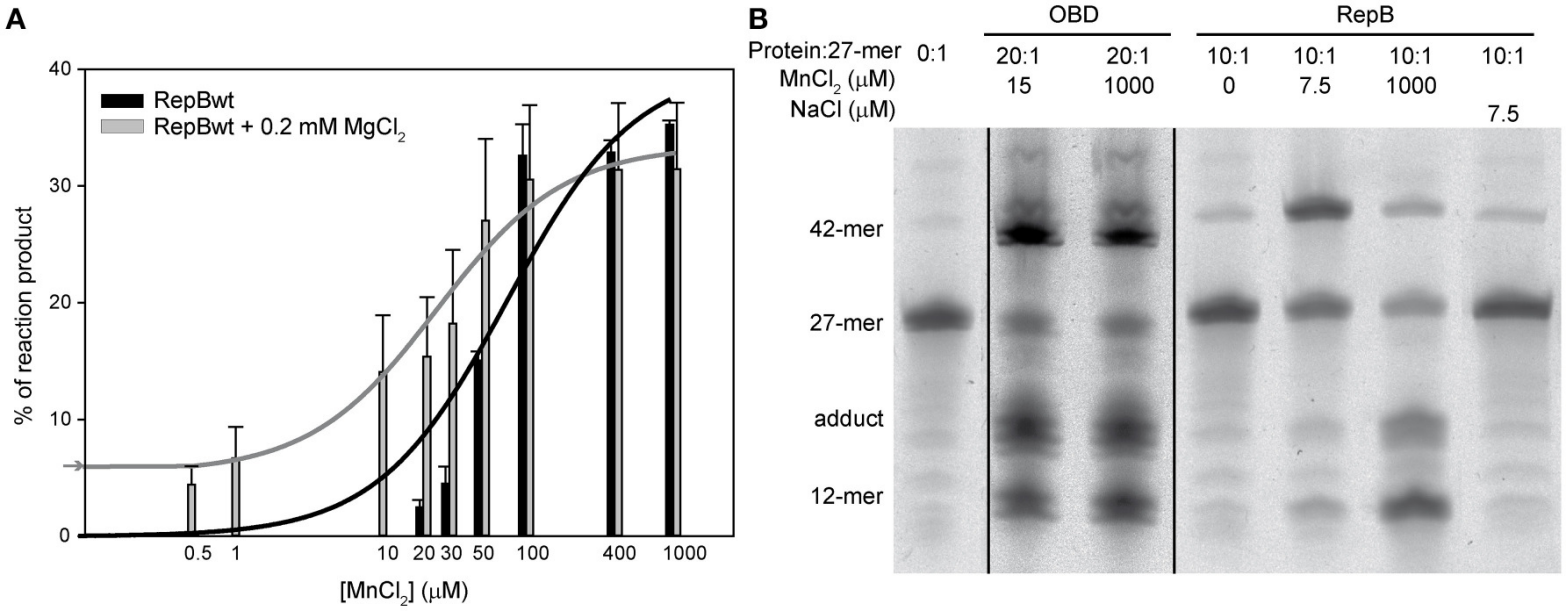
**Nicking and strand-transfer activity of RepB_6_ on ssDNA oligos in the presence of different metal cation concentrations. (A)** The vertical bar graph shows the percentage of reaction products rendered by RepB_6_, in the presence or in the absence of 0.2 mM of MgCl_2_, when the indicated concentrations of MnCl_2_ were added. The 27-mer oligo substrate (500 nM), labeled in 3′ with the fluorescent dye Cy5, and a 10-fold molar excess of the unlabeled 30-mer oligo were incubated with the protein at 37°C for 1 min. The assays were performed at a protein:oligo substrate molar ratio of 1:10. Vertical bars represent the average value of three different measurements and error bars are standard deviations. The activity curves of RepB_6_ supplemented or not with MgCl_2_ were fitted by nonlinear regression (solid lines) to a ligand binding model assuming a single class of binding site for Mn^2+^. The best fit values for the apparent dissociation constant for RepB_6_ was 24.1 ± 6.9 μM in the presence of 0.2 mM of MgCl_2_, and 80.5 ± 26.2 μM in the absence of MgCl_2_. The percentage of reaction products measured in the presence of 0.2 mM of MgCl_2_ with no MnCl_2_ added (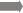) is indicated on the y-axis. **(B)** Reaction product pattern generated by the nicking and strand-transfer activities of RepB_6_ and OBD on ssDNA oligos. The assays were performed as those depicted in **(A)**, at the protein:oligo substrate molar ratio and MnCl_2_ concentration indicated on the top of each lane. As a control, the reaction was also carried out with NaCl instead of manganese salt. The resultant fluorescent oligos were analyzed, visualized and quantified as in Figure 7. To compare the reactions products generated by the activity of OBD and RepB_6_ the images from different gels acquired and processed under the same conditions have been grouped and indicated by dividing lines.

The catalytic activity of RepB_6_ on single-stranded oligos at MnCl_2_ concentrations ranging from 0.1 μM to 1 mM was analyzed using also a RepB protomer:27-mer substrate DNA molecular ratio of 1:10. As for OBD and OBD^D42A^, the catalytic activity of RepB_6_ increased with MnCl_2_ concentration (Figure 8A) and the strand-transfer activity was prevalent under conditions of substrate excess (not shown). By contrary, no product formation was observed in the absence of added Mn^2+^ and RepB_6_ half-maximal activity was reached at ~60 μM of MnCl_2_, a value that is three orders of magnitude higher than that extrapolated from ITC data (*K*_d,37°C_ ≅ 56 nM). To examine the specificity of such high cation concentration requirement for nicking and strand-transfer activities, we measured the activity of RepB_6_ in the same MnCl_2_ concentration range but supplementing the reaction mixture with 0.2 mM MgCl_2_. In the presence of only MgCl_2_, the activity of RepB_6_ became measurable and product formation represented ~5% of the 27-mer added. Moreover, the presence of MgCl_2_ enhanced significantly the activity of RepB_6_ at non-saturating concentrations of MnCl_2_ without varying the maximal activity of the protein (Figure 8A). The increase of RepB_6_ catalytic activity upon Mg^2+^ addition is unlikely to be due to trace amounts of Mn^2+^ in the MgCl_2_ solution, as they should represent less than 4 nM. Besides, the following experimental data suggest that MgCl_2_ does not bind to the active site, although they do not discard that it can partially replace Mn^2+^ in activating nicking and strand-transfer. First, 0.2 mM MgCl_2_ does not stabilize the OBD domain against thermal denaturation in the complete RepB_6_ protein. Second, we have failed to find any evidence of Mg^2+^ high-affinity binding to RepB_6_ through Mg^2+^ direct titration or Mg^2+^/Mn^2+^ competition assays by ITC (not shown). Moreover, Mn^2+^ was the cation found in the active center of the C3 crystals of RepB_6_ even though the crystallization buffer contained 200 mM MgCl_2_ and theoretically lacked Mn^2+^ (Boer et al., 2009).

We have also analyzed the pattern of the reaction products generated by RepB_6_ under conditions of protein excess (10:1 protein:27-mer molar ratio) and observed that it varied depending on the concentration of Mn^2+^ added (Figure 8B). At 7.5 μM MnCl_2_ the main reaction product resulted from the strand-transfer activity of RepB_6_; the observed protein activation relied on the divalent cation as it was not achieved when 7.5 μM NaCl was added instead. Interestingly, at 1 mM MnCl_2_ the proportion of strand transfer product was perceptibly decreased and the reaction was shifted to the formation of nicking product and covalent adduct (Figure 8B). The same effect was achieved by supplementing with 1 mM MgCl_2_, although the advance of the reaction was significantly lower (not shown). By contrast, an excess of OBD protein relative to the 27-mer substrate (molar ratio of 20:1) rendered, both at low and high MnCl_2_ concentration, a pattern of reaction products where the two types of products coexisted (Figure 8B).

## Discussion

### Influence of Mn^2+^ in the structural stability of OBD and RepB_6_

Thermal denaturation of RepB_6_ takes place in two irreversible steps. The first one leads to an inactive form of the protein, and the second one results in protein precipitation (Figure 1). Further characterization of RepB_6_ and of its separate OBD and OD domains showed that the first conformational change exclusively affects the endonuclease domain, impairing its dsDNA binding and ssDNA catalytic activities (Figures 2, 5). The process reflects OBD denaturation, based on DSC data and near-UV CD spectroscopic changes, although in overall RepB the domain secondary structure seems to be largely preserved (Figure 1). The low stability of the OBD domain, whose thermal denaturation takes places with a Tm of 39.5°C, contrasts with the high thermostability of the oligomerization domain, which maintains its native structure at temperatures as high as 80°C (Figure 1 and Supplementary Figure 2).

Mn^2+^ binding results in a strong thermal stabilization of the endonuclease domain, both in its separate form (OBD protein) and in full-length RepB_6_ (Figures 1, 5), which likely correlates with saturation of one high affinity site of RepB per protomer, as measured by ITC (*K*_d_ ~40 nM; Figure 6). Other divalent cations, like Mg^2+^ and Ca^2+^, failed to stabilize RepB_6_ against thermal denaturation and Mg^2+^ binding was not observable by ITC, which pointed to their incapacity to bind RepB_6_ with high affinity. The protective effect of Mn^2+^ binding on the RepB structure likely results from the stabilization of the four protein ligands at the active site (His39, Asp42, His55, and His57; Boer et al., 2009) and of their coordination spheres. Three out of these four ligand residues are linked through several hydrogen bonds. Namely His39 and His55 main chains are interconnected through two H-bonds, whereas the carboxyl group of Asp42 is hydrogen-bonded to the side chains of His55 and Tyr115. Besides, His39 side chain makes a hydrogen bond with the carbonylic oxygen of Leu100, and the carbonylic group of Asp42 is hydrogen-bonded to the main-chain amide-N of Ser44, whose hydroxyl oxygen is connected, in turn, to the main-chain amide-N of Lys50. Additionally, His57 and Ser36 residues form three hydrogen bonds through their main chains and side chains (Supplementary Figure 1). Hence, by stabilizing this network of polar contacts, the Mn^2+^ cation contributes as well to held in place the flexible 21-residue loop that connects strands β2 and β3, and the region comprised between helix α3 and 3_10_-helix η2, both of them flanking the active site (Boer et al., 2009). Moreover, the global conformation of this region might be altered in the metal-free form of OBD^D42A^, thereby explaining the affinity decrease derived from the loss of a metal ligand, as well as the differences found in the enthalpy and entropy of Mn^2+^ binding to OBD^D42A^ with respect to OBD (Figure 6). The D42A variant of OBD not only retains the Mn^2+^ binding capacity but also the catalytic activity, which amounted to ~90% of wild-type OBD under Mn^2+^ saturating concentrations (Figure 7). Therefore, the Asp42 moiety, although not essential for metal binding, contributes significantly to the high affinity of the cation and helps to maintain the architecture of the catalytic groove. Of note, the Tyr115 moiety, hydrogen-bonded to Asp42, is conserved among the Rep proteins of the pMV158 family. The architectural role of this interaction would be also consistent with the lack of nicking activity showed by the Rep protein variant Y116W of pJB01, a *Enterococcus faecium* plasmid belonging to the pMV158 replicon family, which was formely atributed to the involvement of Tyr116 (equivalent to Tyr 115 of pMV158 RepB) in the catalytic reaction (Kim et al., 2006).

Mn^2+^ also binds tightly to other HUH endonucleases like Rep of AAV5, TraI of F, minMobA of R1162, and MobM of pMV158, though with about one-twentieth the affinity for RepB (Hickman et al., 2002; Larkin et al., 2007; Xia and Robertus, 2009; Lorenzo-Díaz et al., 2011). As for OBD^D42A^, Mn^2+^ binding to MobM was enthalpically driven, which indicated that cation binding triggered a conformational rearrangement of MobM structure (Lorenzo-Díaz et al., 2011). Metal-induced stabilization has been proved also in some HUH endonucleases of the Mob class. Mn^2+^ gave the greatest stabilization of minMobA and MobM (Xia and Robertus, 2009; Lorenzo-Díaz et al., 2011), although their protection was significantly lower than that induced in RepB at equal cation concentration.

### Conservation of Mn^2+^ binding traits within the HUH endonuclease superfamily

Configuration of the active site of HUH endonucleases results from the spatial arrangement of a divalent cation and amino acids from several conserved motifs. The presence of an acidic residue involved either directly or indirectly in metal coordination seems to be a common feature in Mg^2+^ or Mn^2+^ binding proteins. Three neutral His side chains coordinating the metal is the configuration most widely conserved among relaxases characterized so far, with the exception of MbeA from plasmid ColE1, with a HEN signature substituting the canonical His triad (Varsaki et al., 2003). Moreover, the interaction through a hydrogen bond between a conserved Asp residue (Asp81) and a His of the 3-His cluster in the active site of relaxase TraI of F seems to do more than orient the His to coordinate the metal. It probably modulates the charge of the His on the metal, allowing a greater polarization of the scisille phosphate bond (Larkin et al., 2007). Substitution of any of the residues of the 3-His cluster by Ala results in no detectable metal binding in TraI of F or minMobA of R1162 (Larkin et al., 2007; Xia and Robertus, 2009). By contrary, the D81A variant of TraI of F binds Mn^2+^ with lower affinity than the wild type enzyme and displays a conditional phenotype, exhibiting minimal activity with MgCl_2_ but wild-type activity with MnCl_2_ (Larkin et al., 2007), which reminds our results for the OBD^D42A^ mutant. In this line, substitution of any of the three His residues of the RepB_6_ metal binding pocket yielded unstable protein variants that precipitated irreversibly upon being overproduced (not shown). In the case of the viral Rep initiators, the His residue which does not belongs to the HUH motif is replaced by an acidic residue. Thus, the metal bound at the active site of Rep of AAV5 is coordinated by two His (89 and 91) and the acidic side chain of Glu82, whose independent substitution results in no detectable binding of Mn^2+^ (Hickman et al., 2002). Similarly, substitution of Glu83 of AAV2-Rep68 (equivalent to Glu82 in AAV5-Rep) by alanine severely impaired the nicking activity of AAV2-Rep68, but residual activity was observed in the presence of Mn^2+^ (Yoon-Robarts and Linden, 2003).

### Role of metal cations on RepB activity

Divalent metals could play a role in the proper positioning of the substrate within the catalytic cavity by neutralizing the charges of the ssDNA substrate. They could help also to orient the catalytic residue/s or enhance the polarization of the scisille phosphate bond. Despite Mn^2+^ high binding affinity, no nicking or strand-transfer activity were detected in RepB_6_ at cation concentrations below 20 μM, which were yet expected to saturate the metal site located at the active center, considering the *K*_d,37°C_ value extrapolated from ITC titration data. Moreover, the concentration of MnCl_2_ required for RepB_6_ half-maximal activity exceeded by three orders of magnitude the *K*_d,37°C_ value (Figure 8). One possibility to explain this apparent discrepancy was that, at these quite low Mn^2+^ concentrations, rejoining of the cleaved 23-mer substrate by full-length RepB_6_ predominated over the strand transfer activity, even when the strand-transfer oligo substrate was in a 10-fold molar excess relative to that harboring the nicking sequence. However, a further increase of the strand-transfer substrate up to a 100-fold molar excess did not increase RepB_6_ catalytic activity (not shown), making this hypothesis unlikely. As such inconsistence did not exist in the OBD^D42A^ mutant and was largely attenuated in the separate endonuclease domain OBD, the distinct protein configuration inherent to each structure might underlie their different behavior. In this context, binding of metal cations to secondary binding sites located at the interface of the RepB_6_ domains and/or protomers, or even between RepB_6_ and the substrate DNA, so that full nicking activity would be reached only when high and low affinity sites become saturated, could explain the apparent inconsistency between the Mn^2+^ binding affinity of RepB_6_ calculated from ITC and the enzymatic assays. In contrast with this, the enhancement of OBD (or OBD^D42A^) activity promoted by Mn^2+^ (Figure 7) most probably derives from the cation binding to the active site.

The structure of the RepB hexamer reveals a high degree of conformational plasticity, allowing differences of up to 55° in the orientation of the OBDs relative to the ODs (Boer et al., 2009, 2016). As a result, RepB_6_ can exists at least in two distinct structural conformations (C2 and C3 structures). The movement of the OBDs and their position relative to the ODs is mainly determined by the flexibility of the hinge region connecting both domains and by the distinct interactions created between the OBD and the hinge region of a protomer and the OD helix α5 of a neighboring protomer (Boer et al., 2016).Interestingly, a divalent cation can bind to this region through the backbone of the hinge region of a protomer and side chains of residues from the own OD and that of an adjacent protomer in an OBD conformation-dependent way. Indeed, null, half or full site occupancy by Mg^2+^ or Ba^2+^ has been observed, respectively, for the inward, intermediate and outward positions of the OBD domains in RepB_6_ C2 structure (Boer et al., 2016). The role of this metal binding site in the orientation of the OBD domains or RepB_6_ activity is presently unknown. However, C2 and C3 structures of RepB_6_ were obtained in crystallization buffers with different divalent metal conditions. So, it is tempting to speculate on the possibility that the metal bound to this second site, which does not exist in separate OBD, could influence the activity of RepB_6_, accounting for both the high cation concentrations required for full activity on ssDNA oligos and the ability of Mg^2+^ to enhance the activity of RepB_6_ at non-saturating concentrations of Mn^2+^ (Figure 8). Characterization of Mob class proteins like MobM or minMobA also suggested the uptake of additional cations for maximal nicking activity (Xia and Robertus, 2009; Lorenzo-Díaz et al., 2011). The OBD movements and the structural elements controlling its relative orientation within RepB_6_ have been suggested to play an important role in the adaptative capacity of RepB to bind diverse DNA structures within the replication origin (Boer et al., 2009). Moreover, the presence of the hinge region in other initiators suggests that it may be a common, crucial structural element for the binding and manipulation of DNA (Boer et al., 2016).

Notably, the pattern of reaction products generated by the activity of RepB_6_ on ssDNA oligos when using an excess of protein respect to the substrate depends on MnCl_2_ concentration. The reaction shifted from favoring formation of the strand transfer product at low (7.5 μM) MnCl_2_ concentration toward formation of the nicking product and the covalent adduct at 1 mM of MnCl_2_ (Figure 8). We hypothesize that a high concentration of divalent metals may reduce the retention of the strand-transfer oligo substrate near the active site of RepB_6_, thereby avoiding the strand transfer reaction. The fact that OBD activity, at the same protein:substrate molar ratio used for RepB_6_, resulted in similar proportions of strand transfer and nicking products, independently of the MnCl_2_ concentration, further support the notion that the path followed by the substrate oligo is different in RepB_6_ and OBD, either due to the presence of the OD domain or to the structural/mechanistic implications of its incorporation into the RepB_6_ hexamer. Interestingly, very high concentrations of MnCl_2_ or MgCl_2_ (≥10 mM) decreased the activity of OBD and RepB_6_ (not shown) probably because they prevented the interaction of the protein with the substrate ssDNA.

### Biological relevance of Mn^2+^ in pMV158 replication

The physiologically relevant metal for Rep and Mob proteins of the HUH endonuclease superfamily is uncertain, as illustrates the variety of metal cations (Mg^2+^, Mn^2+^, Zn^2+^, or Ni^2+^, among others) found in the active site of the HUH endonucleases whose structure has been solved (Hickman et al., 2002; Datta et al., 2003; Boer et al., 2006, 2009; Monzingo et al., 2007; Vega-Rocha et al., 2007; Nash et al., 2010; Francia et al., 2013).

Inside the pMV158 family of replication initiators, information on metal ion recognition has been provided for RepB of pMV158 (this work) and RepB of pJB01 (Kim et al., 2006), which is also active with Mn^2+^. In addition, MobM, the other nucleotidyl-transferase encoded by pMV158, also requires Mn^2+^ for its optimal activity (Lorenzo-Díaz et al., 2011). This paucity of information about the preference for cation usage makes difficult to discern whether the selection of the cation reflects either a preference of the Rep proteins of the pMV158 replicon family or a greater availability of Mn^2+^ in the particular cellular environment. In this sense, inductively coupled plasma mass spectrometry (ICP-MS) analysis revealed milimolar concentrations of cell-associated Mn^2+^ in *Streptococcus pneumoniae* (Jacobsen et al., 2011). Mn^2+^ cations are known to be required *in vivo* for several cellular processes of this bacterium, like capsule formation, metabolism and detoxification, and its cellular homeostasis is maintained even when the extracellular Mn^2+^ is depleted (Jacobsen et al., 2011). Therefore, the availability of such a high concentration of intracellular Mn^2+^ is consistent with the relevance of this cation for certain DNA transactions, such as replication, conjugation or recombination, performed in this bacterium.

## Concluding remarks

Here we report the characterization of the activity and thermal stability of the endonuclease domain of RepB, the initiator protein representative of the pMV158 replicon family of RCR plasmids. RepB is shown to consist of a thermolabile (N-terminal catalytic OBD) and a thermostable (C-terminal hexamerization OD) domain. Binding of Mn^2+^ to the active center of the protein protects the OBD from undergoing a conformational change that implies loss of its tertiary structure and renders the protein both catalytically inactive and unable to recognize the plasmid origin. The Asp42 residue, which is one of the Mn^2+^ ligands in the active center of RepB, was found to be involved in high affinity binding of the divalent cation. Saturation of both the high affinity Mn^2+^ binding site at the active center and the lower affinity additional site(s) seems to be required for maximal activity of full-length hexameric RepB.

## Experimental procedures

### Construction of OBD^D42A^

GeneTailor™ System (Invitrogen) was used to perform site-directed mutagenesis. The mutants were generated by replacement of Asp42 by Ala in the active site of OBD. DNA of plasmid pQE1-OBD (Boer et al., 2009), employed to overproduce OBD, was used as template in the mutagenesis reactions. Overlapping primers were designed following the manufacturer's specifications. The expected mutation was confirmed by DNA sequencing and the resultant mutant was purified as indicated below.

### Protein purification

RepB_6_, OBD, and OD were purified as described previously (Ruiz-Masó et al., 2004; Boer et al., 2009). OBD^D42A^ was purified following the protocol used for the respective wild type form. To study OBD and OBD^D42A^ Mn^2+^-binding affinities by ITC, as well as the effect of Mn^2+^ addition on their catalytic activities, the N-terminal His-tags of OBD and OBD^D42A^ were completely removed by using the exoproteolytic enzymes of the TAGZyme system (Unizyme). Protein concentrations were measured spectrophotometrically using the theoretical molar absorption coefficients. Concentrations of RepB_6_ given throughout the text refer to total protomers.

### Activity of RepB on supercoiled DNA

Mixtures of RepB protein (4 pmol) and pMV158 DNA (0.2 pmol) were incubated in a total volume of 30 μl of buffer B (20 mM Tris-HCl, pH 8.0, 5 mM DTT) supplemented with 100 mM of KCl and different concentrations of MnCl_2_ (ranging from 0.2 to 20 mM) for 30 min at 37°C or 60°C. After incubation, samples were treated with Proteinase K (125 μg/ml) for 10 min at 23°C and mixed with sample loading buffer. Reaction products were analyzed by electrophoresis in 1% agarose gels with 0.5 μg/ml ethidium bromide in TBE buffer. DNA bands were visualized with a GelDoc system (Bio-Rad) and the QuantityOne software (Bio-Rad) was used for the quantitative analysis of the fluorescence intensities given by the different plasmid forms.

### Nicking and strand-transfer activities on single-stranded oligonucleotides

For cleavage and strand-transfer assays, 10 pmol of the 27-mer oligo substrate 5′-TGCTTCCGTACTACG/ACCCCCCATTAA-3′ (where “/” indicates the RepB nick-site) fluorescently labeled with Cy5 were mixed with 100 pmol of an unlabeled 30-mer oligo 5′-TACTGCGGAATTCTGCTTCCATCTACTACG-3′ that provided the 3′-OH substrate for strand transfer, thus avoiding the re-joining of the 27-mer oligo. The mixture was incubated for 1 min at 37°C with RepB_6_ (1 pmol of protomers), OBD, or OBD^D42A^ (1 pmol) in 20 μl of buffer B supplemented with a final concentration of 300 mM NaCl and containing different concentrations of divalent metal salts. Protein samples were previously diluted in 20 mM of Tris-HCl buffer (pH 8.0) supplemented with 430 mM of NaCl and 0.2 mg/ml of BSA. After incubation for 1 min at 37°C, the reaction mixtures were treated with proteinase K (60 μg/ml) and 0.05% of SDS for 10 min at 37°C. Prior to electrophoresis, the samples were mixed with 10 × DNA loading buffer without dye and denatured by heating at 95°C for 3 min. The products were separated on 20% PAA (19:1 acrylamide:bis-acrylamide), 8 M urea denaturing gels. After electrophoresis, the gels were analyzed by using a FLA-3000 (FUJIFILM) imaging system and the QuantityOne software (Bio-Rad) to quantify the reaction products.

For cleavage assays at different temperatures, 3 pmol of the 23-mer oligo substrate 5′-TGCTTCCGTACTACG/ACCCCCCA-3′ (where “/” indicates the RepB nick-site) labeled with ^32^P at the 5′ end using T4 polynucleotide kinase (Sambrook et al., 1989) was incubated for 10 min at 30, 37, and 60°C with different amounts of RepB_6_, ranging from 0.6 to 12 pmol of protomers, in 30 μl of buffer B supplemented with a final concentration of 300 mM NaCl and containing 10 mM of MnCl_2_. After incubation, the reaction mixtures were treated with 60 mM of EDTA and immediately frozen in a mixture of ethanol and dry ice. The reaction products were recovered by ethanol precipitation in the presence of 0.3 M of sodium acetate pH 7. The pellet was washed with 70% ethanol and dissolved in 2 × loading buffer (95% formamide, 100 mM EDTA, 0.5% bromophenol blue, 2.5% xylene cyanol). The samples were denatured by heating at 95°C for 3 min and separated as described above.

### EMSA assays

Reactions to analyze the dsDNA binding capacity of RepB_6_ after being heated or not to 45°C were performed in buffer B supplemented with 300 mM of KCl containing 2.4 μM of RepB_6_ and 0.4 μM of 42-bp oligonucleotide (42-bind) carrying the *bind* locus (coordinates 529–570 of the pMV158 DNA sequence). After 30 min at 25°C, free and bound DNAs were separated by electrophoresis on native 5% PAA gels. The gels were stained with ethidium bromide and the DNA bands were visualized by fluorescence.

### CD assays

CD measurements were performed in a J-810 spectropolarimeter (Jasco Corp.) fitted with a peltier temperature controller, using 1-mm or 10-mm path-length cells for far- and near-UV data acquisition, respectively. To analyze the temperature-associated changes in secondary structure, purified proteins were dialyzed at 4°C against buffer CD (20 mM HEPES, pH 8.0, 4.5% ammonium sulfate, 5% ethylene glycol) containing Chelex-100 (0.14% w/v), and then supplemented with various concentrations of Mn^2+^ by addition of small volumes of concentrated MnCl_2_ stocks prepared in the same buffer. His-tagged OBD and OBD^D42A^ were subjected to an extra-chelating treatment to eliminate trace amounts of divalent metals. Briefly, after purification, the protein samples were incubated with 10-fold molar excess of EDTA for 1 h at 4°C and then dialyzed against buffer CD containing Chelex-100 (0.14% w/v).

CD spectra (average of 4 scans) were acquired using a scan rate of 20 nm min^−1^, a response time of 4 s and a bandwidth of 1 nm. Thermal denaturation experiments were carried out by increasing the temperature from 20 to 95°C at a heating rate of 40°C/h and allowing the cell to equilibrate for 60 s before recording the ellipticity at the selected wavelength. Spectra were recorded in parallel from 20 to 95°C with temperature increments of 10°C, allowing the temperature to equilibrate for 1 min before spectrum acquisition. Buffer contribution was subtracted from the experimental data, and the corrected ellipticity was converted to mean residue ellipticity unless otherwise stated. Data acquisition and processing were carried out using Jasco Spectra-Manager software. Phenomenological description of thermal denaturation profiles was carried out by means of Equation (1) using the Origin software (Microcal Inc.):

(1) $$\begin{array}{l} \Theta \;=\;\Theta_{D}(T)\;-\;[\Theta_{D}(T)\;-\;\Theta_{N}(T)]/\{1\\ \;+\text{ exp}[A(T\;-\;T_{1/2})/RTT_{1/2}]\} \end{array}$$

where Θ_*D*_(*T*) and Θ_*N*_(*T*) are the ellipticities values of the denatured and native states of the protein at the absolute temperature *T, T*_1/2_ is the half-transition temperature, *R* is the gas constant, and *A* accounts for the transition cooperativity. Θ_*D*_(*T*) and Θ_*N*_(*T*) values in Equation (1) were approximated as linear functions of *T* (Ruiz et al., 2014).

### Calorimetric studies

Mn^2+^ binding to RepB, OBD and OBD^D42A^ was studied at 25°C by ITC using a VP-ITC microcalorimeter (GE Healthcare, Madrid, Spain). Before measurements, the proteins were exhaustively dialysed at 4°C against buffer ITC (20 mM HEPES, pH 7.6, 400 mM KCl) containing Chelex-100 (0.14% w/v) and MnCl_2_ solutions were prepared in the final dialysate after removing Chelex-100. Titrations were performed by stepwise injection of 1 mM MnCl_2_ solution into the reaction cell loaded with the protein at concentrations of 95–119 μM. Typically, 13 × 7 μl injections followed by several 15 μl injections were performed for RepB_6_ and OBD, and 27 × 10 μl for OBDD^42A^, while stirring at 307 rpm. The heat of MnCl_2_ dilution was determined in separate runs and subtracted from the total heat produced following each injection. The experiments were carried out at 25°C. Data acquisition and analysis were carried out using the ITC-Viewer and Origin-ITC softwares (GE Healthcare). Mn^2+^ dissociation constants at 37°C were extrapolated from ITC data by means of the van't Hoff equation assuming that binding occurred without heat capacity change.

DSC measurements were performed at a heating rate of 60°C/h in a VP-DSC microcalorimeter (MicrocaI Inc.), at a constant pressure of 2 atm. RepB was equilibrated in CD buffer supplemented with the required Mn^2+^ concentration. Microcal DSC-Viewer and Origin-DSC software was used for data acquisition and analysis. Excess heat capacity functions were obtained after subtraction of the buffer-buffer base line and transformed into molar heat capacities dividing by the number of moles of RepB in the DSC cell.

## Author contributions

JR and LB purified RepB and OBD. MS cloned and purified the mutant OBD^D42A^. MM and JR performed the CD and calorimetric assays and analyzed the data. JR performed the protein activity assays with supercoiled plasmid DNA. JR and LB performed the protein activity assays with single stranded oligos. JR, MM, and GdS designed the study and wrote the article. All authors discussed the results, edited, and approved the manuscript.

## Funding

This work was supported by grants from the Spanish Ministry of Economy and Competitiveness (BFU2015-70052-R to MM; BFU2010-19597 to GdS, AGL2012-40084-C03 and AGL2015-71923-REDT to GdS). Additional funding to MM was provided by the CIBER de Enfermedades Respiratorias (CIBERES), an initiative of the Instituto de Salud Carlos III (ISCIII).

### Conflict of interest statement

The authors declare that the research was conducted in the absence of any commercial or financial relationships that could be construed as a potential conflict of interest.
