# Supplementary material for: Metal-Induced Stabilization and Activation of Plasmid Replication Initiator RepB
Source: Front Mol Biosci. 2016 Sep 21;3:56. doi: 10.3389/fmolb.2016.00056 (PMC5030251; doi:10.3389/fmolb.2016.00056)
Supplement: Supplementary file 1 [file DataSheet1.docx]

Supplementary Material

Metal-induced stabilization and activation of plasmid replication initiator RepB

**José A. Ruiz-Masó^1^, Lorena Bordanaba-Ruiseco^1^, Marta Sanz^1^, Margarita Menéndez^2,3,*^ and Gloria del Solar^1,*^**

^1^Centro de Investigaciones Biológicas (CSIC), ^2^Instituto de Química-Física Rocasolano (CSIC) and ^3^Ciber of Respiratory Diseases, Madrid, Spain.

* Correspondence: Margarita Menéndez, Instituto de Química-Física Rocasolano (CSIC), Serrano, 119, 28006 Madrid, Spain, [mmenendez@iqfr.csic.es](mailto:mmenendez@iqfr.csic.es); and Gloria del Solar, Centro de Investigaciones Biológicas (CSIC), Ramiro de Maeztu 9, 28040 Madrid, Spain, [gdelsolar@cib.csic.es](mailto:gdelsolar@cib.csic.es).

Supplementary Figures


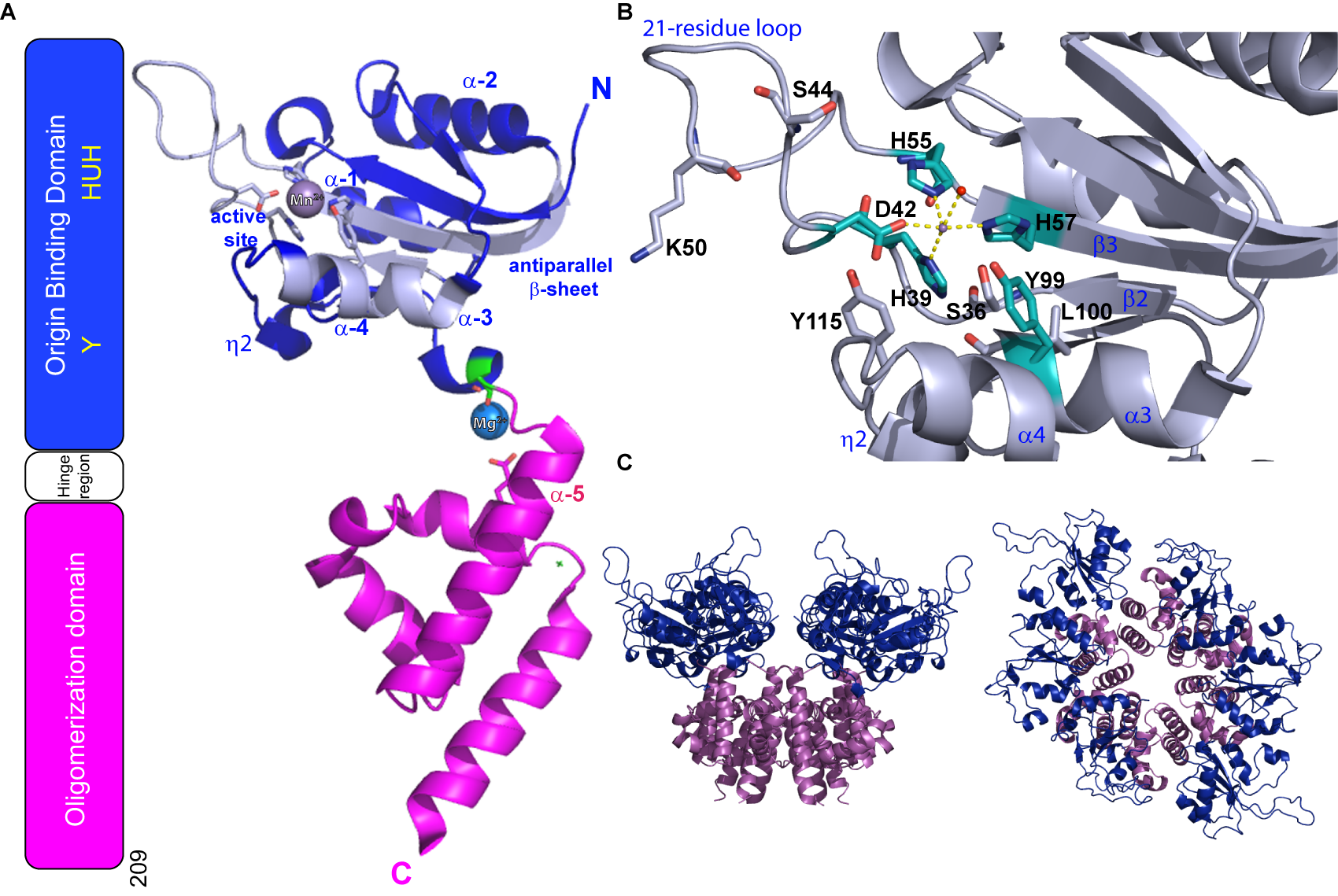


**Supplementary Figure 1**. **Structure of RepB and of the active center.** **A)** A cartoon drawing of a single protomer of RepB with the OBD domain in the outward position. The OD domain and the hinge region are also indicated. The active site and the Mn^2+^ ion (magenta sphere) are indicated using a stick representation of cation binders at the active site. The same representation was used to indicate the residues involved in the coordination of Mg^2+^ (blue sphere) in the hinge region (PDB code: 4U87). The location of the helices α-1to α-5, and the 3_10_-helix η2 flanking the antiparallel β-sheet is also indicated. B) A close-up of the active site. The Mn^2+^-binding residues (cyan) as well as the residues of the network of polar contacts likely stabilized by Mn^2+^ coordination (light blue) are indicated using a ball-and-stick representation. C) Cartoon representation of the C-2 structure of the RepB hexamer in different orientations with the OBD and OD domains colored in blue and magenta, respectively.


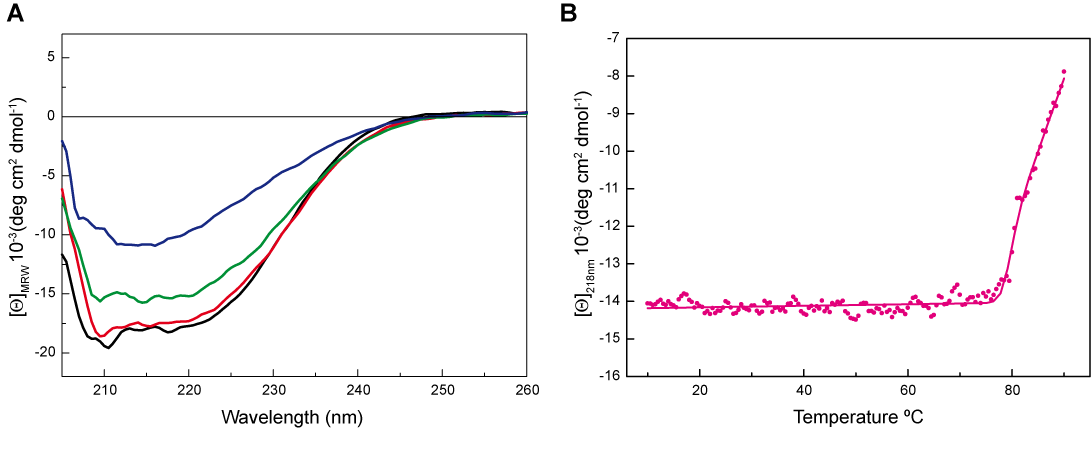


**Supplementary Figure 2**. **Temperature-induced changes in the secondary structure of separate OD.** **A)** Far-UV CD spectra of OD (20 µM) at 30 ºC (▬), 70 ºC (▬), 80 ºC (▬) and 90 ºC (▬). B) Temperature transition curve of OD (20 µM) measured by CD at 218 nm ([Θ] represents the mean residue ellipticity).


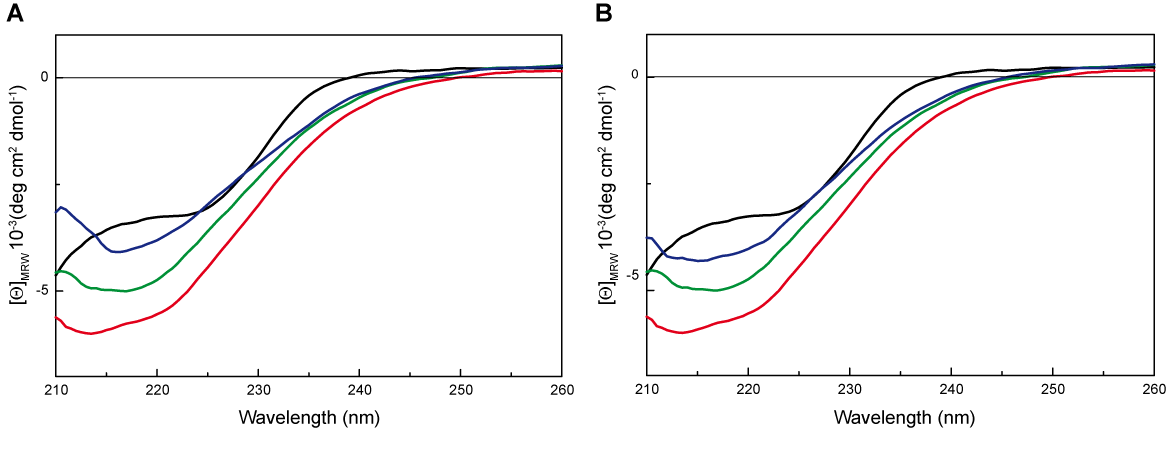


**Supplementary Figure 3**. **Temperature-induced changes in the secondary structure of OBD and OBD^D42A^.** The figure shows the far-UV CD spectra of (A) OBD (19 µM) and (B) OBD^D42A^ (19 µM) at 20 ºC (▬), 60 ºC (▬), 70 ºC (▬) and 80 ºC (▬). [Θ] represents the mean residue ellipticity.
